# Hyporesponsive fetus-specific T cell responses in multiparous human pregnancy

**DOI:** 10.3389/fimmu.2025.1634430

**Published:** 2025-07-29

**Authors:** Jessica C. Morgan, Grace E. Hynes, Jared M. Pollard, Michael S. Andrade, Jong Cheol Jeong, Anita S. Chong

**Affiliations:** ^1^ The University of Chicago, Department of Obstetrics and Gynecology, Division of Maternal Fetal Medicine, Chicago, IL, United States; ^2^ The University of Chicago, Department of Surgery, Section of Transplantation, Chicago, IL, United States

**Keywords:** maternal-fetal tolerance, T cell tolerance, multiparous pregnancy, pregnancy, ELISpot assay, fetus-specific responses

## Abstract

Pregnancy acts as both a tolerogenic and sensitizing event, inducing T cell hypofunction and humoral sensitization. Mouse studies reported that T cell hypofunction is a key mechanism in preserving fetal viability, but this phenomenon remains uncharacterized in humans. In this study, we developed an assay to specifically assess fetal-specific T cell tolerance in uncomplicated, full-term human pregnancies. The majority of maternal PBMCs stimulated with matured fetus-matched dendritic cells (CBDCs) exhibited low IFNγ responses that were significantly lower than third-party fetus-unmatched CBDCs. This hyporesponsiveness to matched CBDCs extended to the production of a range of Th1 and Th2 cytokines, and was not associated with increased immunoregulatory cytokines, IL-10 or IL-1RA. Unexpectedly, a small number of grand multiparous individuals displayed heightened IFNγ responses to fetus-matched CBDCs; these individuals also exhibited heightened Th1 and Th2 cytokine responses. Together, our study introduces a novel assay to measure fetal antigen-specific T cell responses that confirmed T cell hypofunction as an immunological mechanism enabling successful pregnancy in humans in addition to mouse. It also implicates alternative tolerance mechanisms that allow successful pregnancies to proceed even in the presence of fetus-specific T effector cell reactivity. These findings have implications for understanding immune dysregulation in pregnancy complications and for improving transplantation outcomes in multiparous women.

## Introduction

Pregnancy induces a paradoxical immune state where fetus-specific T cell tolerance induced systemically or locally within the placental interface is necessary for successful semi-allogeneic pregnancies (1–5). T cell intrinsic hypofunction and expansion of regulatory T cells have been identified to play critical roles in mouse models of semi-allogeneic pregnancy (6–13). Furthermore, in the absence of pregnancy-experienced B cells and fetus-specific antibodies, pregnancy-induced T cell tolerance is persistent and mediates the spontaneous acceptance of fetus-matched heart allografts transplanted at post-partum day 45-60 (14). While the presence of B cells and fetus-specific antibodies do not antagonize the development of pregnancy-induced T cell hypofunction or Treg expansion, pregnancy-experienced B cells or fetus-specific antibodies overrides post-partum T cell tolerance to transplanted fetus-matched hearts (14). Those findings provided an unexpected link between pregnancy-induced T cell tolerance and fetus-matched organ transplantation that potentially has clinical consequences for post-partum candidates for living-related organ transplantation.

Observations of pregnancy-induced T cell tolerance mouse models raise the question of whether T cell tolerance towards the allogeneic fetus is replicated in humans because this is salient for parsing T cell responses in post-partum transplant candidates. Furthermore, humans harbor higher frequencies of alloreactive memory T cells than laboratory mice, which can be generated not only by direct exposure to alloantigens via blood transfusions, transplantation and pregnancy, but also by heterologous immunity that generates cross-reactive memory T cells upon exposure to environmental antigens and infections (15–17). Thus, it is likely that some mothers harbor substantial frequencies of memory T cells specific for fetal HLA antigens, which have to be constrained to attain complicated full-term pregnancy. Indeed, we recently reported that memory CD8 T cells can be reprogrammed by pregnancy towards dysfunctional states with downregulated effector memory and enriched for exhaustion transcriptional programs (6). If constraint of memory T cell tolerance is a conserved phenomenon in human pregnancy, it is possible that insights into the mechanisms of pregnancy-induced tolerance for both naive and memory T cells can be leveraged to improve transplant outcomes in the clinic.

Obstetric complications are not uncommon in the general population, with incidence of preeclampsia estimated at 3-10%, preterm birth at 10-12%, miscarriage during the first trimester at ≤20%, and recurrent pregnancy loss effecting <5% of pregnancies (18–21). Each of these diagnoses carry significant physical and emotional burden on patients, and the underlying etiology for each of them is not well understood. The notion that there is an immunological basis for some, if not all, of these conditions is broadly accepted (22–25), and identifying therapeutic targets for these conditions would have significant benefit maternal, fetal, and neonatal outcomes. We hypothesize that abnormal or high-risk pregnancies may, at least partially, be due to inefficient programming of fetus-specific T cell hypofunction compared uncomplicated full-term pregnancies.

The aim of this pilot study was to develop an assay to evaluate whether fetus-specific T cell hypofunction is observed in uncomplicated full-term pregnancies in humans. To this end, we quantified IFNγ production by maternal PBMCs in response to ex vivo matured fetus-matched cord blood dendritic cells (CBDCs). Our study demonstrated that the majority of index pregnancies did not result in sensitization, and in fact, were significantly reduced compared to responses to third-party CBDCs. Additionally, we identified a small subset of full-term pregnancies in grand multiparous women with significantly elevated responses to matched compared to unmatched CBDCs. These latter observations are consistent with a second immune checkpoint facilitating successful pregnancies in women with high frequencies of fetus-specific IFNγ-producing T cells.

## Results

### Intrapartum IFNγ responses to fetus-derived DC stimulation

**Figure 1:**
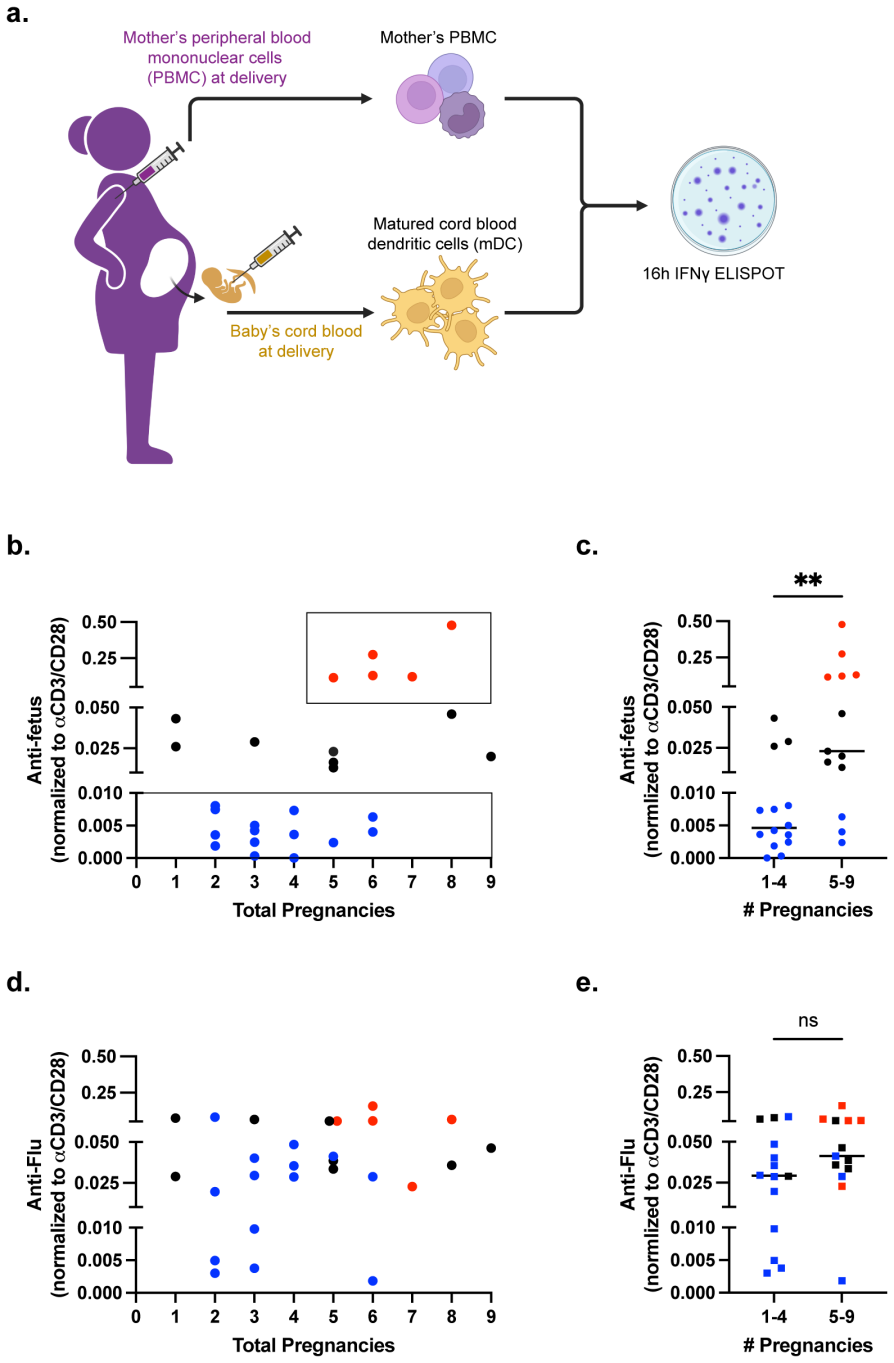
Majority of pregnancies result in minimal fetus-specific IFNγ responses. **(a)** Pictorial summary of experimental workflow. n=27 samples of maternal PBMCs were stimulated with matched DCs (mDCs) in an IFNγ ELISPOT assay. **(b)** Scatter plot showing anti-fetus IFNγ response, normalized to anti-CD3/CD28 stimulation, relative to the number of pregnancies reported. **(c)** Scatter plot comparing anti-fetus IFNγ response binned into groups with 1-4 or 5-9 pregnancies. Statistical significance determined by Mann-Whitney test. **(d)** Scatter plot showing normalized anti-influenza (Flu) IFNγ response. **(e)** Scatter plot comparing anti-influenza IFNγ response binned into groups with 1-4 or 5-9 pregnancies. Individual responders are color coded as low (blue), mid (black), and high (red) in **(b–e)**. Statistical significance determined by Mann-Whitney test. All data are represented as individual values with mean ± standard deviation (SD) shown. **P < 0.01. ns not significant (P>0.05).

Numerous studies have reported that semi-allogeneic pregnancy in mice does not result in expanded populations of fetus-specific IFNγ-producing T cells, contrasting with semi-allogeneic graft rejection which stimulates strong IFNγ responses (7, 14, 26). To test whether IFNγ hyporesponsiveness is observed in human pregnancy, maternal PBMCs were collected from 27 participants on the day of, but prior to, delivery, and frozen until use in stimulation assays. (**Table 1**). These thawed PBMCs were stimulated with cord blood-derived fetal dendritic cells (CBDCs), which were generated with a 5-week ex vivo expansion and maturation protocol into CD11c^+^ CD14^+^ CD1a^+^ HLA-DR^+^ DCs (27; **Supplementary Figure S1a**). Maternal IFNγ responses to matched fetus-derived CBDCs (mDCs) were quantified with an IFNγ ELISpot assay (**Figure 1a**). Additional culture conditions included media only, stimulation with anti-CD3/CD28, stimulation with unmatched CBDCs (uDC), and influenza vaccine in the presence or absence of mDCs or uDCs (**Supplementary Figure S1b**). Spot counts were normalized and presented as a percentage of spot counts observed with anti-CD3/CD28 stimulation, to address potential heterogeneity in the quality of collected PBMCs (**Figure 1b**). Spot counts were binned into low, medium and high responses, with an average of <0.5, <2.5 and >20% of the anti-CD3/CD28 responses respectively (**Supplementary Figure S2a**).

**Table 1:** Patient demographics binned into higher (high + mid) vs low IFNγ responders.

		Low	Mid/High	P Value
		(n=14)	(n=13)	
Race				
	Black	9	9	1.000
	White	1	4	0.165
	Hispanic	3	0	0.222
	Asian	1	0	1.000
Avg. Age		34.643	34.307	0.987
Reproductive Events				
	Avg. Gravidity	3.5 ± 1.204	5.308 ± 2.534	0.015
	Avg. Parity	2.786 ± 1.342	3.308 ± 1.888	0.259
	Avg. Full-term	2.5 ± 1.401	2.615 ± 2.016	0.778
	Avg. Preterm	0.286 ± 0.633	0.692 ± 0.947	0.296
	Avg. Abortion	0.714 ± 0.825	2 ± 1.581	0.017
Influenza Vaccine (FLUARIX QUADRIVALENT 2023/2024 Formula)				
	# Yes	4	4	1.000
	# No	5	8	0.257
	# Unknown	5	1	0.165
Delivery Type				
	Vaginal	2	2	1.000
	Cesarean	10	11	0.648
	Not Listed	2	0	0.481
Avg. Gestational Age (days)		271 ± 7.603	260 ± 14.686	0.053
Avg. Baby Weight (grams)		3170 ± 454.600	2894.5 ± 693.800	0.149

P values were calculated using Welch’s t-test (gestational age, baby weight) and Fisher’s exact test (race, reproductive events, influenza vaccine, and delivery type subcategories). *P < 0.05; **P < 0.01; ***P < 0.001.

Approximately 52% of antepartum PBMCs obtained at parturition displayed low responses to fetus-matched DCs (**Figure 1**; **Supplementary Figure S2a**), including some individuals where the index pregnancy investigated in this study was the 4-6th pregnancy. This observation supports the conclusion that human pregnancy induces minimal sensitization of fetus-specific T cells. A second cohort of PBMCs (29.6% of samples) had intermediate responses. Notably, a third cohort of high responder PBMCs (18.5% of samples) was observed. This high responder cohort comprised a subset of grand multiparous women wherein the index pregnancy was ≥ 5th pregnancy (**Figure 1c**). High IFNγ responses did not correlate with the number of parity (pregnancies up to 20 weeks), pre-term pregnancy, full-term pregnancy, or abortions (**Supplementary Figures S3a–j**). When the 27 individuals were binned into higher and lower responders, higher IFNγ responses were significantly associated with number of gravidity and abortion (**Table 1**).

Variable levels of IFNγ responses to matched fetal antigens could reflect baseline differences between individual PBMC samples. To test this possibility, we interrogated IFNγ recall responses to influenza, which was also binned into <0.5, <2.5 and >20% of the anti-CD3/CD28 responses (**Figure 1d**; **Supplementary Figure S1b**). The majority of individuals exhibited intermediate responses (14 of 27), while 29.6% (8 of 27) had high and 18.5% had low responses. The majority of low responders to mDCs had intermediate responses to influenza (8 of 14, 57.1%), and there was no statistical difference in influenza response between individuals where the index pregnancy was less than versus greater than 5 pregnancies (**Figure 1e**).

### Intrapartum IFNγ responses to matched versus unmatched fetus-derived DCs

**Figure 2:**
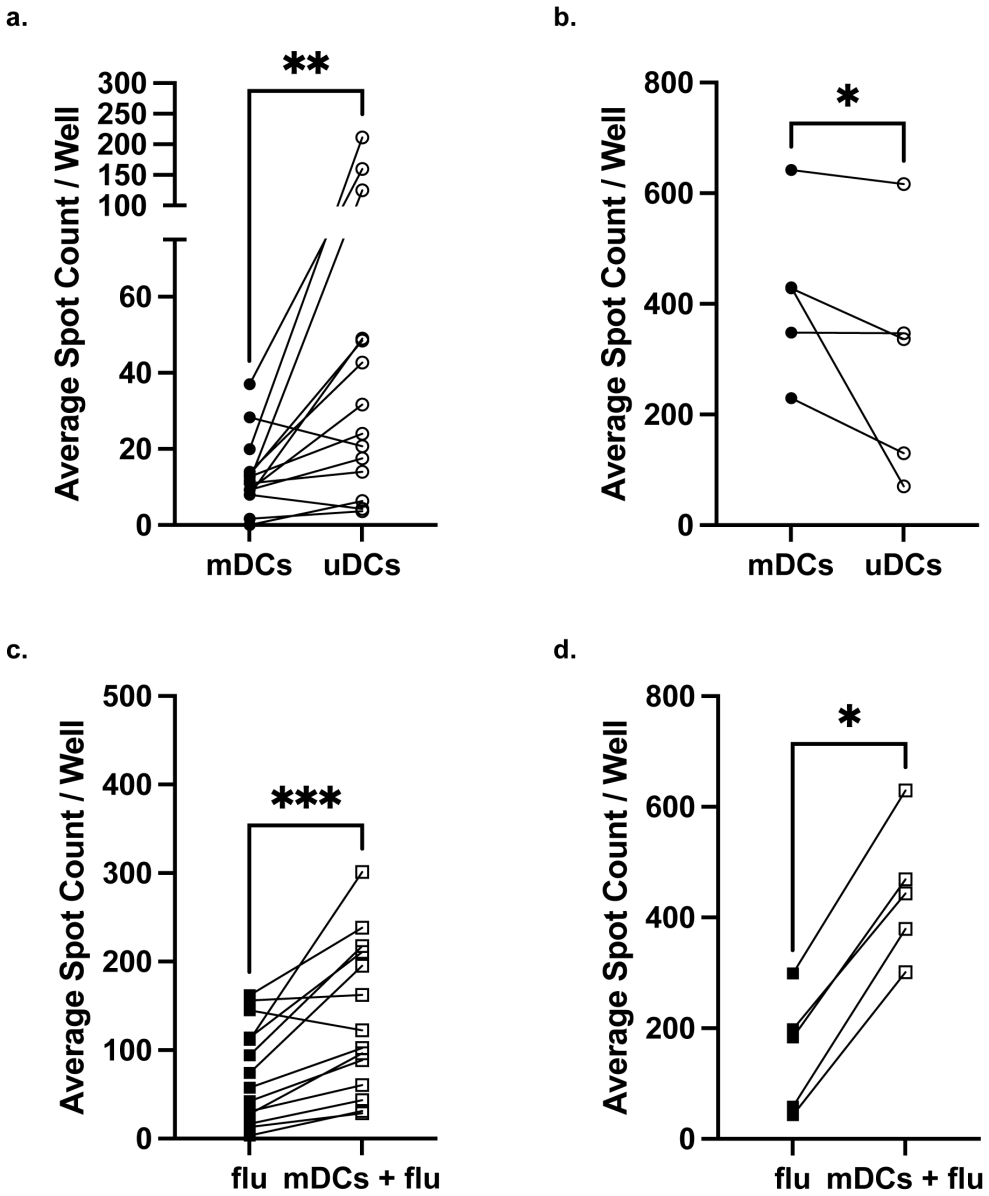
Maternal IFNγ responses to matched compared to unmatched fetal dendritic cells. Comparison of IFNγ response between mDCs and uDCs in low responders **(a)** or high responders **(b)** identified in **Figure 1b**. Comparison of IFNγ response by low responders **(c)** or high responders **(d)** stimulated with influenza (Flu) alone and with influenza+mDCs. Statistical significance was determined using the Wilcoxon matched-pairs signed rank test. *P < 0.05; **P < 0.01, ***P < 0.001. All data are represented as individual PBMC responses.

We noted that 4 of 5 of the highest responders were also the highest responders to influenza, thus prompting us to compare their IFNγ responses to third-party unmatched CBDCs (uDCs) that were randomly selected (**Supplementary Figures S2c, d**). Similar to responses to influenza vaccine, the majority (63.0%) of PBMCs exhibited intermediate responses while 22.2% exhibited low responses. Four PBMC samples were high responders to uDCs, mDCs, and influenza, prompting us to perform matched pair analysis for responses to mDCs versus uDCs (**Figure 2**). For the low responders to mDCs, the IFNγ response to uDCs was significantly higher suggesting a hyporesponsive state specifically for fetal antigens (**Figure 2a**). In contrast, high responders to mDCs had significantly reduced responses to uDCs, supporting the notion of sensitization to fetal antigens in these individuals (**Figure 2b**).

To address the concern that mDCs for the low responders were poor antigen-presenting cells, and the PBMC samples simply had poor viability, we quantified IFNγ responses to influenza vaccine in the presence of mDCs. We reasoned that since mDCs are haplo-identical to maternal PBMCs, they would be able to present influenza-derived peptides on maternal HLA and thus enhance IFNγ responses. Indeed, low responders exhibited a significantly enhanced IFNγ response when stimulated with mDCs and influenza vaccine compared to vaccine alone (**Figure 2c**). High responders also exhibited significantly stronger IFNγ responses when stimulated with influenza antigen and mDCs, compared to influenza alone (**Figures 2b, d**). Collectively these observations support the conclusion that intrapartum PBMCs are specifically hyporesponsive to fetal antigens in a majority of pregnancies, and that some full-term successful pregnancies may be sensitizing, especially in grand multiparous women and/or individuals with globally high IFNγ responses.

### Fetus-specific Th1 and Th2 cytokine responses are reduced in low IFNγ responders

**Figure 3:**
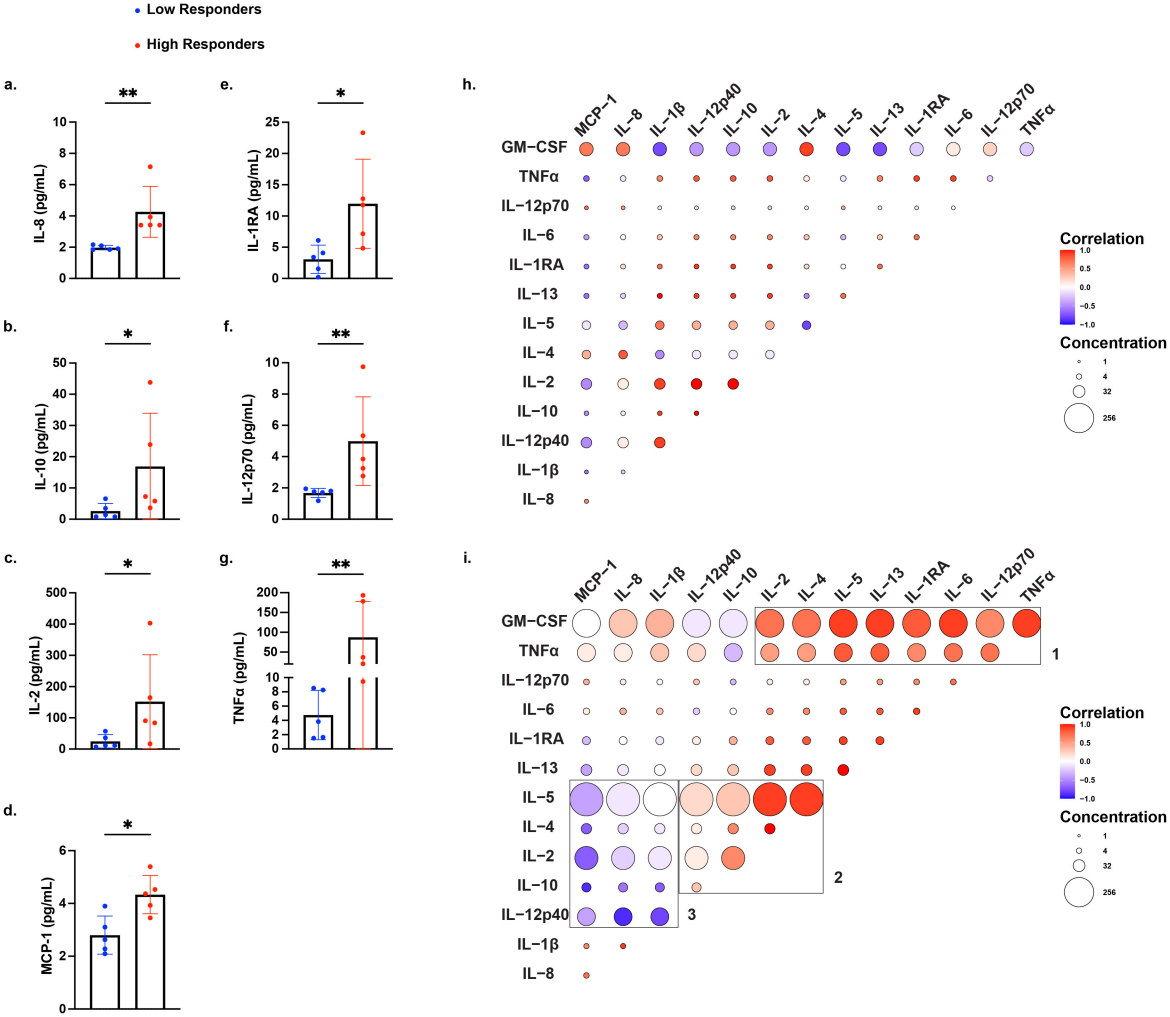
Differential production of proinflammatory and regulatory cytokines by high and low IFNγ producers stimulated with mDCs. Cytokine production by all 5 high IFNγ responders and 5 matched low IFNγ responders, identified in **Figure 1b**, using a multiplex assay to quantify 14 cytokines: IL-8, IL-1β, IL-12p40, IL-10, IL-2, IL-4, IL-5, IL-13, IL-1RA, IL-6, IL-12p70, TNFα, GM-CSF, and MCP-1. Statistical significance was determined using the Mann-Whitney test, comparing normalized fold increase in cytokine levels for **(a)** IL-8, **(b)** IL-10, **(c)** IL-2, **(d)** MCP-1, **(e)** IL-1RA, **(f)** IL-12p70, and **(g)** TNFα. Each data point represents the average of duplicate wells for each individual. Bars represent mean ± standard deviation (SD). *P < 0.05; **P < 0.01. Spearman correlation matrices of cytokine responses in low responders **(h)** and high responders **(i)** after stimulation with mDCs. Positive correlations are shown in red, and negative correlations are shown in blue, with color intensity corresponding to the strength of correlation and circle size corresponding to the average concentration of the cytokine. For **(h)** and **(i)**, can the font size be enlarged for figure legends for Correlation and Concentrations?

To test whether low IFNγ producers have a distinct, potentially immunoregulatory, cytokine profile, and whether high IFNγ responders produce a broader array of pro-inflammatory cytokines, we analyzed the supernatants from the IFNγ ELISPOT assay using a 13-cytokine multiplex ELISA assay. To minimize confounding variables, all 5 high IFNγ responders to mDCs and 5 low responders analyzed were matched by maternal age, race/ethnicity, and gravidity. Overall, when compared to low responders, high responders to mDCs co-produced significantly more IL-8, IL-10, IL-2, MCP-1, IL-1RA, IL-12p70, and TNFα. A trend toward significantly increased levels of IL-5 and IL-13, which could reach statistical significance with larger sample numbers (**Figures 3a–g**; **Supplementary Figures S4a, b**). In contrast, both groups produced comparable amounts of IL-4, IL-6, IL1β, IL-12p40, and GM-CSF (**S4c–g**). Notably, low IFNγ producers did not produce more of the immunomodulatory cytokines, IL-10 or IL-1RA. These observations indicate that high responders generate a more robust cytokine response to mDCs that was characterized by proinflammatory and regulatory cytokines, while low responders had an overall reduced ability to produce these cytokines.

Spearman’s correlation analyses revealed distinct patterns in cytokine co-production between low and high responders (**Figures 3h, i**). In low responders, raw concentrations of all cytokines were reduced compared to high responders, consistent with a global hyporesponsiveness to mDC stimulation, rather than regulation by immunosuppressive cytokine production. Cytokine production by high responders clustered in three distinct modules: 1) GM-CSF and TNFα expressing module consistent with a proinflammatory Th1 signature, 2) a Th2 and regulatory cytokine anchored by IL-2, IL-4, and IL-5 expression, and 3) an innate inflammatory module marked by high production of MCP-1, IL-8, and IL-1β that was negatively correlated with the cytokines IL-5, IL-4, IL-2, IL-10, and IL-12p40. Together, these data highlight a dynamic cytokine profile in high responders spanning effector, helper, and regulatory cytokine networks, consistent with robust T cell activation and broad immune system engagement.

## Discussion

Our study quantifying maternal responses using matched vs unmatched CBDCs allows for a rigorous test of whether the fetus-specific T cell hyporesponsiveness observed in mouse semi-allogeneic pregnancy is recapitulated in humans. Unlike mice that are maintained in specific pathogen free facilities and have a limited repertoire of memory alloreactive T cells, humans accumulate an increasing repertoire of memory alloreactive T cells with age, primarily through heterologous immunity in healthy individuals (15, 16, 28). The ability to quantify fetus-specific IFNγ responses at parturition allowed us to stringently test whether pregnancy is a non-sensitizing event or alternatively, whether uncomplicated full-term pregnancy can proceed despite high frequencies of circulating fetus-specific IFNγ-producing T cells.

A majority of prior studies assessing fetus-specific T cell responses have used paternal PBMCs as stimulators (29). While this approach is valid for studies on pregnancy in inbred mouse strains, in human studies, this approach does not account for the possibility of incorrectly assuming paternity. More importantly, even when paternity is correctly assigned, this approach does not represent a truly fetus-specific response, as it includes maternal responses to non-inherited paternal alloantigens. To circumvent these shortcomings, we used cord-blood stimulators which ensures that the maternal T cell response to only fetal antigens associated with the index pregnancy is specifically assessed. However, cord blood has a lower frequency of DCs than adult peripheral blood, is less stimulatory *in vitro*, and evokes lower incidence of graft-versus-host disease during transplantation compared to adult marrow or peripheral blood transplants (30, 31). To address the possibility that immature cord blood mononuclear cells are poor T cell stimulators, we subjected cord blood cells to a ~5-week maturation process to generate CBDCs that were potent stimulators in IFNγ ELISPOT assays (27).

The majority (52%) of matched maternal-fetal dyads with normal index pregnancy exhibited minimal IFNγ responses to fetal stimulators, and this response was significantly reduced compared to responses to third-party CBDCs. Matched CBDCs were not inherently non-stimulatory as they enhanced maternal IFNγ responses to influenza vaccine, by processing and presenting influenza antigens on inherited maternal HLA. Low responders ranged from mothers with 2-9 pregnancies, notwithstanding the caveat that paternity of prior pregnancies relative to index pregnancy was unverified. This hyporesponsiveness was not limited to the IFNγ response, but extends across a range of Th1 and Th2 cytokines. Importantly, this was not correlated with increased expression of anti-inflammatory cytokines, such as IL-10 and IL-1RA, which were also minimally produced. Taken together, these data support prior mouse studies and the hypothesis that the majority of allogeneic pregnancies in humans are immunologically non-sensitizing, resulting in minimal maternal T effector cell generation against fetal antigen.

Unexpectedly, a subset of dyads with normal index pregnancy exhibited heightened IFNγ responses to mDCs compared to uDCs. These data represented grand multiparous individuals with ≥ 5 births with ≥20 weeks gestation. We noted that 4 of 5 high responders to mDCs also had highest responses to influenza and uDCs, raising the possibility that these individuals were intrinsically hyperresponsive. Indeed, analysis of 13 cytokine responses revealed that high responders co-produced a wide array of cytokines across effector, helper, and regulatory axes. Whether this hyperresponsiveness to mDCs is due to sensitization by pregnancy, pre-existing heterologous immunity, intrinsically heightened ability to produce cytokines, or some combination of the above, requires a larger cohort study as well as complementary studies in mice. Regardless of etiology, the heightened fetus-specific responses in a subset of uncomplicated pregnancies suggest the existence of alternative mechanisms to successfully constrain these potentially pathogenic responses in pregnancy. Indeed, our recent studies in mouse support this possibility by demonstrating that pre-existing memory fetus-specific CD8+ T cells acquire an exhausted phenotype and transcriptome during pregnancy (26).

Limitations of our study include the low sample size, inability to ascertain if the index pregnancy shared the same paternity with prior pregnancies, and variability in quality of cord blood and maternal blood collection. Other inherent limitations of studying human pregnancy are their heterogenous nature including fetal sex or magnitude of HLA mismatch, and occurrences of major and minor complications throughout pregnancy, at birth and in the postpartum period. We attempted to control for these limitations with our inclusion and exclusion criteria, which necessitated that the patient have no history of autoimmune disease, organ transplant, or blood transfusion during the pregnancy. However, given the diverse patient population that had higher rates of obstetric and medical complications than the general population, some variables were not possible to control for. Another limitation is that this study was performed only in patients who were delivering liveborn neonates in the third trimester, which we designated as relatively “healthy” pregnancies. Our findings are therefore useful in generating hypotheses to test their role in abnormal pregnancies such as those ending in early pregnancy loss, intrauterine fetal demise, or even neonatal death.

A key strength of our study is the representation of patients who identify as Black or African American, a population historically underrepresented in immunological and pregnancy-related research. There was also a wide range of maternal age, gravidity, and parity, which added a broad clinical context to our findings. The majority of samples were collected prior to scheduled cesarean delivery, which reduced the chances of immunologic changes that are known to occur during labor and facilitated ease of sample collection especially of the umbilical cord blood.

This study is novel in design and incorporation of fetus-derived dendritic cells to stimulate cytokine responses to measure of maternal T cell response to fetal antigens in uneventful full-term human pregnancy. We succeeded in supporting our hypothesis that the maternal T cell response is minimally induced or possibly even suppressed in the majority of pregnancies. An unexpectedly strong T cell response was observed in a subset of individuals with grand multiparity, yet this response did not prevent progression of an uncomplicated, full-term pregnancy. Further research is needed to evaluate specific maternal-fetal immune responses in additional cohorts of uneventful pregnancies, those with high gravidity and parity, and where index pregnancies are impacted by preeclampsia, miscarriage, or late-term fetal demise. Nevertheless, by assessing the full extent of T cell responses induced by pregnancy in humans, this research carries promise in its application towards improving pregnancy outcomes, as well as transplantation outcomes in postpartum women.

## Materials and Methods

### Enrollment criteria

Pregnant patients older than 18, at any gestational age, gravidity, and parity that consented for fetal cord blood collection were considered for inclusion. Patients were excluded if they had a diagnosed autoimmune or acquired immune disease, personal history of organ or tissue transplant, and/or a blood transfusion during current the pregnancy. Medical charts were abstracted for pregnancy outcomes and complications, which included parity, history of health problems and birth outcomes, body mass index prior to pregnancy, and diagnoses of diabetes, hypertension, preeclampsia, and infectious diseases during current pregnancy. Delivery data included method of delivery and duration of labor, as well as fetal sex, weight, length, and gestational age.

### Isolation of peripheral blood mononuclear cells

Maternal peripheral blood was collected at the time of admission for a scheduled cesarean delivery, or before the onset of active labor, after obtaining informed written consent from the patient. This time coincided with routine collection of blood from patients to reduce the number of blood draws patients had to undergo. Peripheral blood mononuclear cells (PBMCs) were isolated from whole blood by density gradient centrifugation using Ficoll-Paque™ Plus (Fisher Scientific, Cat. #45001752), washed three times with PBS, resuspended at 10 × 10^6^ cells/mL and cryopreserved in CryoStor CS10 medium (Stemcell Technologies, Cat. #07930). PBMCs were stored in liquid nitrogen until use in the ELISPOT assays without additional *in vitro* culture or expansion.

### Isolation of CD34+ progenitor cells from cord blood mononuclear cells

Cord blood was collected from consenting mothers after delivery of neonate and separation from the umbilical cord. About 30-40 mL of umbilical cord blood was collected and cord blood mononuclear cells (CBMCs) were isolated using Ficoll-Paque™ Plus (Fisher Scientific, Cat. #45001752). Following centrifugation, the CBMCs were washed, resuspended at 10 × 10^6^ cells/mL and cryopreserved in CryoStor CS10 medium (Stemcell Technologies, Cat. #07930).

### Maturation and expansion of CD34+ progenitor cells

Maturation and expansion of CD34^+^ progenitor cells were performed as described in Bedke et al. (43). In brief, thawed CBMCs were suspended in 100ul of PBS containing 2% FBS and 1mM EDTA. CD34^+^ cells were enriched with an EasySep Human CD34 positive selection kit II (Stemcell Technologies, Cat #17856) and washed five times with 2% FBS. CD34^+^ progenitor cells were used for generating cord blood dendritic cells (DCs) or were cryopreserved in CryoStor CS10 medium for later use.

CD34^+^ progenitor cells were resuspended at 5 × 10^4^ cells/mL in FS36 culture media (Iscove’s Modified Dulbecco’s Medium (Cytiva, Cat. #SH30228.01) with 10% heat inactivated FBS, 5 units/mL Penicillin G Sodium, 50 ug/mL streptomycin sulphate, 0.1 mM β-mercaptoethanol) and supplemented with 25 ng/mL Fms-related tyrosine kinase 3-Ligand (Flt3-L), 10 ng/mL Stem cell factor (SCF), 10 ng/mL interleukin-3 (IL-3), 10 ng/mL interleukin-6 (IL-6)). Cells were cultured in a 24-well plate for 7 days, then harvested and centrifuged at 300 xg for 10 minutes at room temperature. Cells were counted and re-seeded at 5 × 10^4^ cells/mL in FS36 culture media, and this was repeated on day 14. On day 21, cells were harvested and re-seeded at 2 × 10^5^ cells/mL in FTS culture media supplemented with 25 ng/ml Flt3-L, 10 ng/mL SCF, 10 ng/mL Thrombopoietin (TPO) for another 7 days. On day 28, cells were harvested, seeded into a 6-well plate at 1 × 10^6^ cells/mL in IMDM medium and incubated for 10 minutes. Non-adherent cells were removed by washing with IMDM two times, and fresh FS36 supplemented with 20 ng/mL IL-4 and 50 ng/mL GM-CSF was added to the adherent cells and cultured for another 5–6 days. Cells were fed every other day by semi-depletion with fresh IMDM supplemented with IL-4 and GM-CSF.

### Characterization of matured cord blood dendritic cells

Fresh cultured or thawed CBDCs were thawed for phenotypic analysis. Cells were washed twice at 350 x g for 5 minutes in 2 ml of 1x PBS and resuspended in 1x PBS at a concentration of 10 x 10^6 cells/ml. BD Horizon FV575V stock (stock was prepared following the manufacturer’s instruction and stored at -20C freezer; Cat. #565694) was added to the cell suspension at 1:1000 dilution and incubated for 10 minutes at room temperature in the dark. Cells were washed twice at 350 x g for 5 minutes in 2 ml of BD staining buffer (Cat. #554656) and resuspended in the BD staining buffer at a concentration of 10 x 10^6 cells/ml. 100 ul of cells were aliquoted into the FACS staining tube and an incubated with the following mAbs: CD14-BV421 (HCD14, Cat. #325628, BioLegend), CD11c-BB515 (3.9, Cat. #301618, BioLegend), CD34-PerCP-eFluor710 (4H11, Cat. #46-0349-42, Invitrogen), CD1a-PE (HI419, Cat. #300106, BioLegend), CD33-APC (P67.6, Cat. #366606, BioLegend), HLA-DR-APCCy7 (L243, Cat. #307618, BioLegend). Cells were stained for 15 minutes at 4°C in the dark following manufacturer’s instruction. The stained cells were then washed twice at 350 x g for 5 minutes in 2ml of BD staining buffer and resuspended for flow cytometry. Samples were acquired on the BD LSR Fortessa at the Cytometry and Antibody Technology Core Facility of the University of Chicago. The data was analyzed using FlowJo (Version 10.10.0).

### ELISpot assay

ELISpot plates (Millipore Sigma MSIPS4W10) were pre-wet with 15ul of 35% ethanol, washed two times with 200ul of PBS, and coated with anti-human IFNγ mAb (Mabtech, Cat. #3420-3-1000) capture antibody overnight at 4 ˚C. PBMCs and matured DCs in DMEM culture media (DMEM with 10% heat inactivated FBS, 5 units/mL Penicillin G Sodium and 50 ug/mL streptomycin sulphate) were plated at 2.5 × 10^5^ cells/well. Influenza vaccine (FLUARIX QUADRIVALENT 2023/2024 Formula 0.5ml, lot: HL72T) was used at 1:40 dilution. Positive control wells were PBMC (2.5 × 10^4^ cells/well) stimulated with anti-CD3 (Mabtech, Cat. #3605-1-50) and anti-CD28 (Mabtech, Cat. #3608-1-50) mAbs at 1:1000 dilution. After overnight culture, the supernatants were removed and frozen at -20 ˚C. ELISPOT plates were washed with PBS and incubated sequentially with biotinylated anti-human IFN-γ mAb (Mabtech, Cat. #3420-6-1000), streptavidin-HRP (Mabtech, Cat. #3310-9-1000) and ready-to-use TMB substrate (Mabtech, Cat. #3651-10). The reaction was quenched by rinsing the plate under running tap water, and then air-dried overnight in the dark.

ELISpot plates were scanned and analyzed using a CTL S6 ELISpot reader (Cellular Technology, Ltd). Samples were plated in triplicates for each experimental condition, and the spot counts per sample were averaged for analysis.

### Multiplex cytokine assay

Supernatants from the ELISpot assay were thawed at 4˚C, centrifuged at 3000xg for 10 minutes to remove debris. Supernatant from the following conditions were sent for analysis: media, matched DCs (mDCs), influenza, and influenza+mDCs. Five high responder samples were diluted with DMEM culture media ether 2- or 4-fold depending on spot counts while low responder samples were analyzed undiluted. Duplicate samples were shipped to Eve Technologies (Calgary, Alberta, Canada) for multiplex cytokine analysis. The following cytokines were tested: GM-CSF, IL-1β, IL-1RA, IL-2, IL-4, IL-5, IL-6, IL-8, IL-10, IL-12p40, IL-12p70, IL-13, MCP-1, and TNFα.

All analysis of cytokine data used average concentration values, with the “media” experimental condition averaged as negative controls. Data in **Figure 3**; **Supplementary Figure 2** were calculated as average fold increase from negative controls.

### Sex as a biological variable

As this is a study on human pregnancy, we included only one sex (those assigned female at birth) in our study population. Sex was not explicitly considered as a biological variable in regard to interpretation or analysis of results.

### Statistics

Statistics were performed using GraphPad Prism (version 10.3.1). P values comparing multiple group were calculated using Kruskal-Wallis 1-way ANOVA with Dunn’s *post hoc* test. P values comparing two experimental groups were calculated using a Mann-Whitney test. P values comparing experimental conditions within the same sample were calculated using a Wilcoxon matched-pairs signed rank test. Correlation matrices were generated using Spearman’s correlation coefficient, r. Statistical tests and significance thresholds are indicated in the legend of each figure.

### Study approval

This study and consent documents were approved by the University of Chicago Institutional Review Board (IRB #21-1486). Informed written consent was obtained from all patients prior to their inclusion in the study for the collection of their own peripheral blood as well as umbilical cord blood after delivery of the neonate.

## Data Availability

The original contributions presented in the study are included in the article/**Supplementary Material**. Further inquiries can be directed to the corresponding author/s.

## Supplementary material

Supplementary Figure 1 Maturation of fetal dendritic cells and ELISpot assay conditions. **(A)** Gating strategy for matured cord blood dendritic cells (CBDCs). Bottom row displays fetal DCs after the 4-week maturation protocol. **(B)** Scatter plot showing average spot count per well for all conditions, including media only (M), anti-CD3/CD28, matched dendritic cells (mDCs), unmatched dendritic cells (uDCs), influenza (flu) alone, influenza (flu) + mDCs, and influenza (flu) + uDCs.

Supplementary Figure 2 IFNγ response to mDCs, influenza and third-party uDCs. **(A)** Bar plot summarizing IFNγ responses from **Figure 1b**, and **(B)** **Figure 1**, categorized into low, mid, and high responder groups as in **Figure 1b**. **(C)** Scatter plot showing IFNγ response to 3^rd^ party uDCs, normalized to anti-CD3/CD28 stimulation, relative to the number of pregnancies reported. Individual responders were color coded as in **Figure 1b**. **(D)** Scatter plot comparing anti-3rd party IFNγ response binned into 1-4 or 5-9 pregnancies. Statistical significance determined by Mann-Whitney test. *P < 0.05. P values (in **(A, B)**) were determined by Kruskal-Wallis 1-way ANOVA with Dunn’s *post hoc* test (*P < 0.05; **P < 0.01, ***P<0.001, ****P<0.0001) or unpaired t-test (^##^P < 0.01). All data are represented as individual PBMC responses with mean ± standard deviation (SD) shown.

Supplementary Figure 3 Magnitude of fetus-specific IFNγ response T cells is not correlated with number of parity, full-term or pre-term pregnancies, or abortions. Scatter plot and bar plots showing anti-fetus IFNγ response, normalized to anti-CD3/CD28 stimulation, grouped by total parity **(A, D)**, full-term pregnancy **(B, E)**, preterm pregnancy + abortions **(C, F)**, preterm pregnancy **(G, I)**, or abortions **(H, J)**. Responders are color coded as in **Figure 1b**. P values were determined by Kruskal-Wallis 1-way ANOVA with Dunn’s *post hoc* test. All data are represented as individual PBMC responses with mean ± standard deviation (SD) shown.

Supplementary Figure 4 Comparably produced cytokines by high and low IFNγ producers stimulated with mDCs. Fold increase in cytokines produced by PBMC from 5 high IFNγ responders and 5 low IFNγ responders. **(A)** IL-5, **(B)** IL-13, **(C)** IL-4, **(D)** IL-6, **(E)** IL-1β, **(F)** IL-12p40, and **(G)** GM-CSF. Statistical significance was determined using the Mann-Whitney test. Each data point represents the average of duplicate wells for each individual, with mean ± standard deviation (SD) shown.
