# Supplementary figures and images for: Hyporesponsive fetus-specific T cell responses in multiparous human pregnancy
Source: Front Immunol. 2025 Jul 29;16:1634430. doi: 10.3389/fimmu.2025.1634430 (PMC12339333; doi:10.3389/fimmu.2025.1634430)

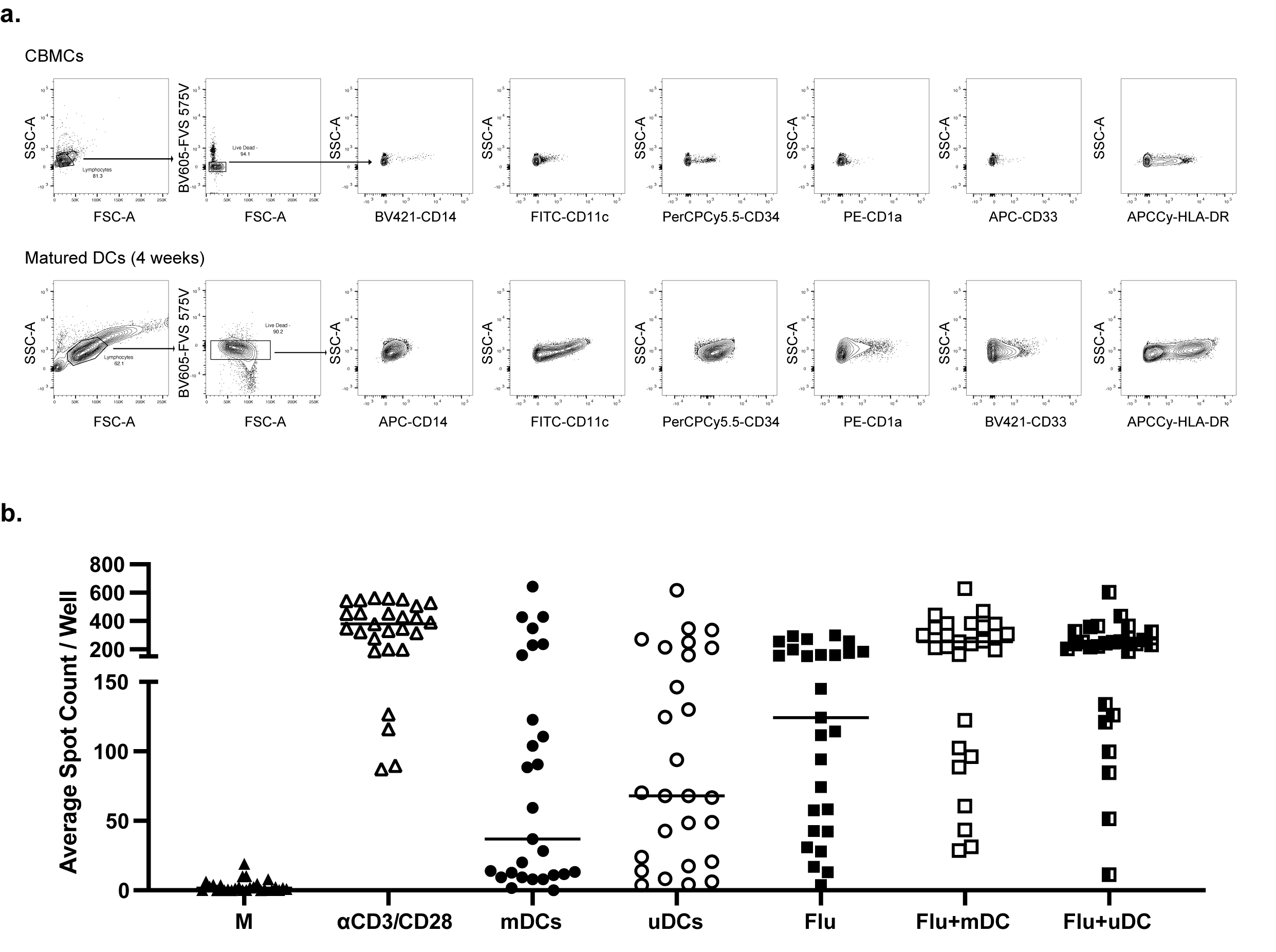

Supplement: Supplementary Figure 1 — Maturation of fetal dendritic cells and ELISpot assay conditions. (A) Gating strategy for matured cord blood dendritic cells (CBDCs). Bottom row displays fetal DCs after the 4-week maturation protocol. (B) Scatter plot showing average spot count per well for all conditions, including media only (M), anti-CD3/CD28, matched dendritic cells (mDCs), unmatched dendritic cells (uDCs), influenza (flu) alone, influenza (flu) + mDCs, and influenza (flu) + uDCs. [file Image1.tif]

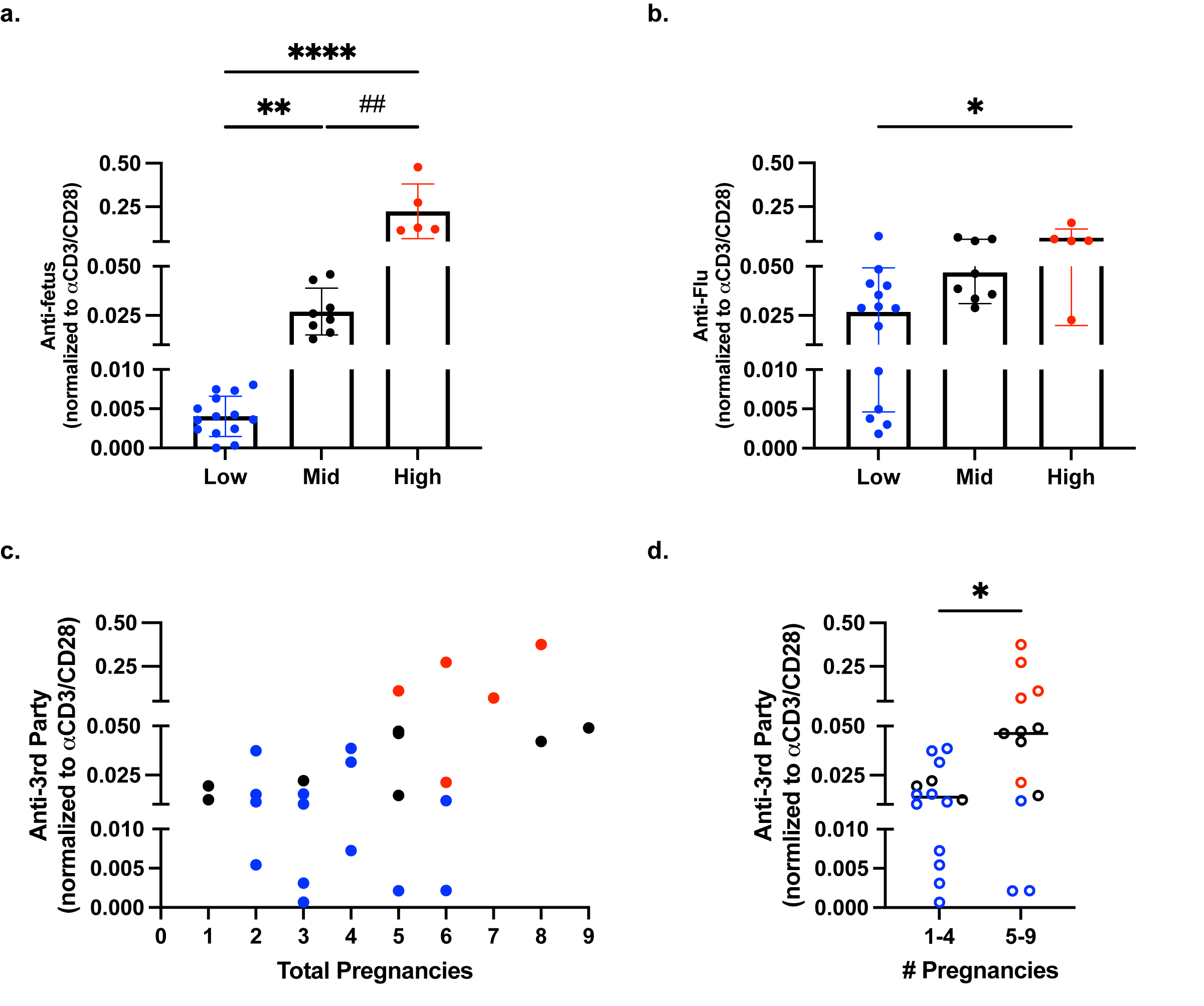

Supplement: Supplementary Figure 2 — IFNγ response to mDCs, influenza and third-party uDCs. (A) Bar plot summarizing IFNγ responses from Figure 1b , and (B) Figure 1 , categorized into low, mid, and high responder groups as in Figure 1b . (C) Scatter plot showing IFNγ response to 3rd party uDCs, normalized to anti-CD3/CD28 stimulation, relative to the number of pregnancies reported. Individual responders were color coded as in Figure 1b . (D) Scatter plot comparing anti-3rd party IFNγ response binned into 1-4 or 5-9 pregnancies. Statistical significance determined by Mann-Whitney test. *P < 0.05. P values (in (A, B)) were determined by Kruskal-Wallis 1-way ANOVA with Dunn’s post hoc test (*P < 0.05; **P < 0.01, ***P<0.001, ****P<0.0001) or unpaired t-test (##P < 0.01). All data are represented as individual PBMC responses with mean ± standard deviation (SD) shown. [file Image2.tif]

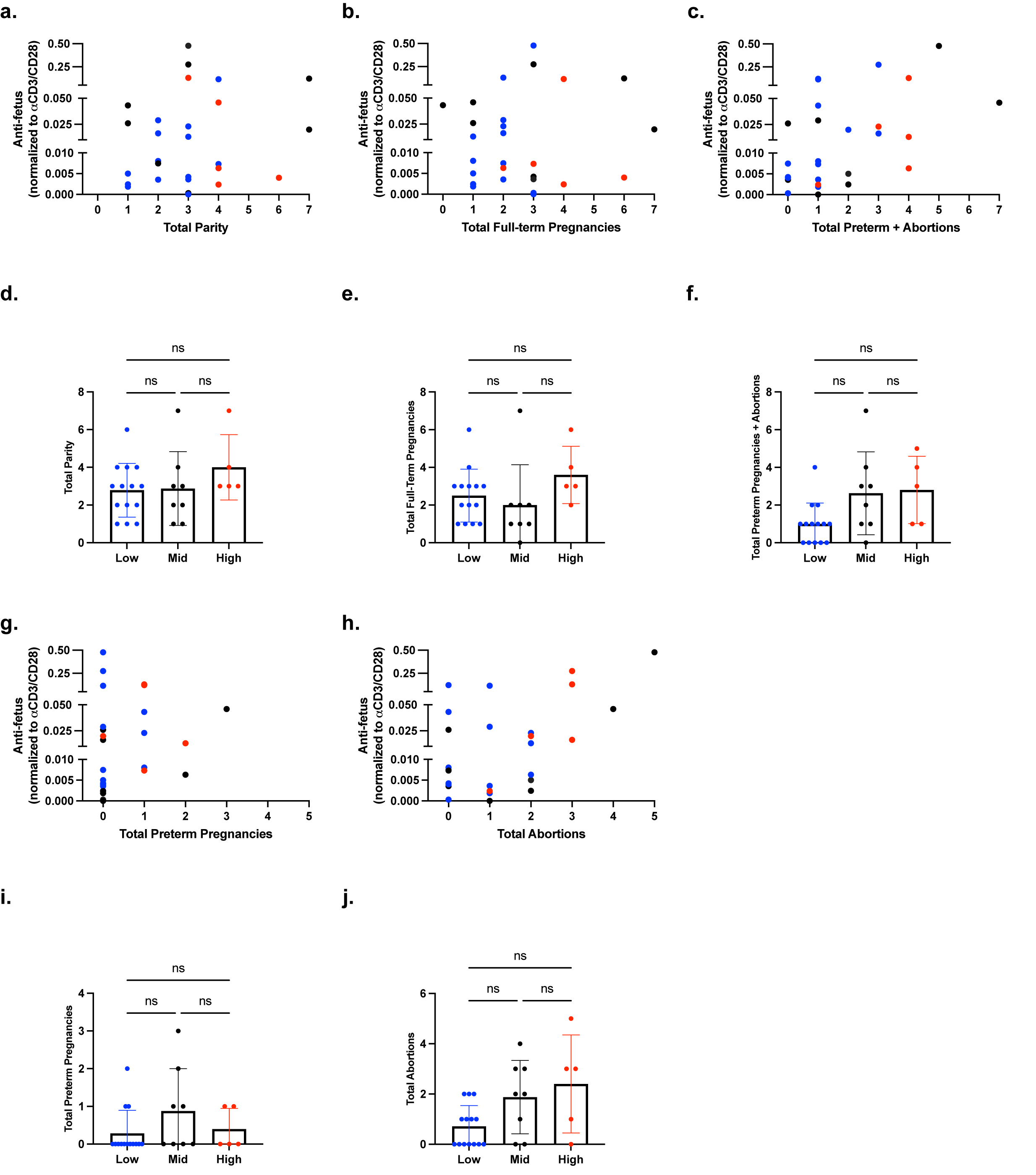

Supplement: Supplementary Figure 3 — Magnitude of fetus-specific IFNγ response T cells is not correlated with number of parity, full-term or pre-term pregnancies, or abortions. Scatter plot and bar plots showing anti-fetus IFNγ response, normalized to anti-CD3/CD28 stimulation, grouped by total parity (A, D), full-term pregnancy (B, E), preterm pregnancy + abortions (C, F), preterm pregnancy (G, I), or abortions (H, J). Responders are color coded as in Figure 1b . P values were determined by Kruskal-Wallis 1-way ANOVA with Dunn’s post hoc test. All data are represented as individual PBMC responses with mean ± standard deviation (SD) shown. [file Image3.tif]

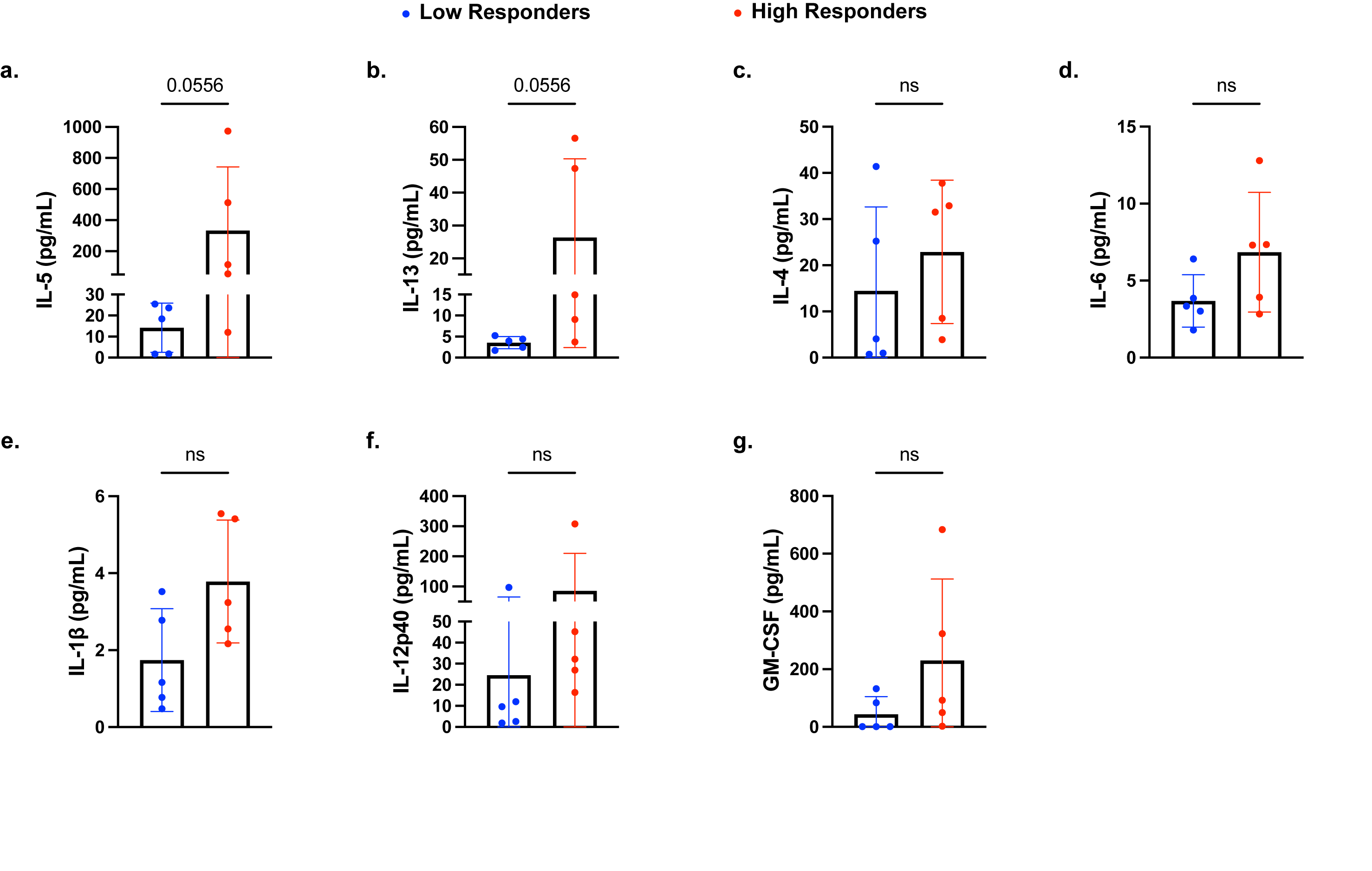

Supplement: Supplementary Figure 4 — Comparably produced cytokines by high and low IFNγ producers stimulated with mDCs. Fold increase in cytokines produced by PBMC from 5 high IFNγ responders and 5 low IFNγ responders. (A) IL-5, (B) IL-13, (C) IL-4, (D) IL-6, (E) IL-1β, (F) IL-12p40, and (G) GM-CSF. Statistical significance was determined using the Mann-Whitney test. Each data point represents the average of duplicate wells for each individual, with mean ± standard deviation (SD) shown. [file Image4.tif]
